# Development and Evaluation of Hydrogel-Based Sulfasalazine-Loaded Nanosponges for Enhanced Topical Psoriasis Therapy

**DOI:** 10.3390/ph18030391

**Published:** 2025-03-10

**Authors:** Sunil Kumar, Anroop B. Nair, Varsha Kadian, Pooja Dalal, Babu Lal Jangir, Bandar Aldhubiab, Rashed M. Almuqbil, Ahmed S. Alnaim, Nouf Alwadei, Rekha Rao

**Affiliations:** 1 Department of Pharmaceutical Sciences, Guru Jambheshwar University of Science and Technology, Hisar 125001, India; sunilkundu450@gmail.com (S.K.); kadyanvarsha313@gmail.com (V.K.); lamba808@gmail.com (P.D.); 2 Atam Institute of Pharmacy, Om Sterling Global University, Hisar 125001, India; 3 Department of Pharmaceutical Sciences, College of Clinical Pharmacy, King Faisal University, Al-Ahsa 31982, Saudi Arabia; baldhubiab@kfu.edu.sa (B.A.); ralmuqbil@kfu.edu.sa (R.M.A.); asaalnaim@kfu.edu.sa (A.S.A.); nalwadei@kfu.edu.sa (N.A.); 4 Department of Pharmacy, School of Health Sciences, Sushant University, Gurugram 122003, India; 5 Department of Veterinary Pathology, College of Veterinary Science, Lala Lajpat Rai University of Veterinary and Animal Sciences, Hisar 125004, India; drbabu.jangir@gmail.com

**Keywords:** sulfasalazine, cyclodextrins, melt method, oxidative stress, orthokeratosis, anti-psoriatic activity

## Abstract

**Background:** The low solubility and poor skin permeability of sulfasalazine (SLZ) present significant challenges for its effective topical delivery. The objective of the current investigation is to formulate a hydrogel-based SLZ-loaded cyclodextrin nanosponge for topical therapy in psoriasis. **Methods:** SLZ-loaded nanosponges were prepared by the melt polymerization method and evaluated for physiochemical characteristics, drug release, and cytocompatibility. The selected nanosponges (SLZ-NS4) were transformed to hydrogel and further evaluated for rheology, texture, safety, skin permeability, and in vivo for anti-psoriatic effect in mouse tail and imiquimod-induced psoriasis-like inflammation models in mice. **Results:** Physiochemical data confirms nanoscale architecture, drug inclusion in nanosponges, crystalline structure, and formulation stability. The release profile of SLZ-NS4 revealed sustained release behavior (22.98 ± 2.24% in 3 h). Cytotoxicity assays indicated negligible toxicity against THP1 cells, resulting in higher viability of cells than pure SLZ (*p* < 0.05). The HET-CAM assay confirmed the safety, while confocal laser scanning microscopy demonstrated deeper skin permeation of SLZ. In the mouse tail model, a remarkable decline in relative epidermal thickness, potential improvement in percent orthokeratosis, and drug activity with respect to control was observed in animals treated with SLZ-NS4 hydrogel. The efficiency of the developed SLZ-NS4-loaded hydrogel in treating psoriasis was confirmed by the decline in PASI score (81.68 ± 3.61 and 84.86 ± 5.74 with 1 and 2% *w*/*v* of SLZ-NS-HG). Histopathological analysis and assessment of oxidative stress markers revealed the profound anti-psoriatic potential of the fabricated SLZ-NS4 hydrogel. **Conclusions:** These findings highlight the profound potential of the developed delivery system as an effective topical therapy for psoriasis.

## 1. Introduction

Psoriasis, an autoimmune ailment, primarily influences skin with varying severity. It is associated with highly inflamed erythematous with scaly lesions. These lesions are the results of the abnormal proliferation of cells of the epidermis, their incomplete cornification, and the preservation of the nucleus in cells of the *Stratum corneum*. In this skin disorder, histopathological modifications occur, including epidermal hyperplasia with differentiation of keratinocytes, enhanced angiogenesis, and significant inflammatory infiltrates [1]. As per the literature reports, about 0.1–4% of children and 0.5–11.4% adult population suffer from psoriasis [2]. According to the 2024 Global Atlas Report, high-income nations are seeing a particularly noticeable rise in psoriasis prevalence [3].

The most prominent treatment options for psoriasis include systemic therapy, phototherapy, and topical therapies [4]. The chronic and recurrent nature of this ailment necessitates greater doses of drugs, which in turn could result in additional side effects in systemic therapy. Further, another problem associated with this disease is long-term therapy, which generally leads to withdrawal of treatment by patients, thereby worsening their conditions. Nevertheless, topical treatments play a significant role in mild to severe psoriasis as they can overcome systemic and phototherapy issues [5]. The topical route also helps to target the skin disorder locally without harming other tissues. In addition, topical therapy is employed as a supporting modality with systemic therapy in severe cases of this skin disease. Indeed, the dose of the systemic agent or phototherapy can be reduced with the use of topical adjunct therapy. Hence, topical therapy wherein skin targeting is involved becomes a key choice for the management of psoriasis and associated symptoms [6,7,8]. On the other hand, the *Stratum corneum*, which has a multilamellar lipidic structure, is a major barrier that drugs may only partially overcome [9]. Various drug factors like molecular weight, solubility, lipophilicity, and pKa largely influence the rate of skin penetration [10]. On the other hand, drug delivery using nanocarriers, polymer/drug complexes, or ion pairs generally results in a drug reservoir in the skin [11]. In addition, studies have shown that topical therapy with nanocarriers has the potential to improve drug delivery to the skin layers, hence improving psoriasis treatment efficacy [12,13,14]. These carriers are capable of localizing in the skin layers and providing sustained drug release to the inflammatory and hyperproliferative regions.

Sulfasalazine (SLZ) is an anti-inflammatory and immunomodulatory agent, which is formed by a combination of the 5-aminosalicylic acid and sulfapyridine through azo bonds [15]. Additionally, SLZ possesses anti-bacterial action [16]. In psoriasis, it acts by suppressing the immune system via reducing immunoglobulin action [17]. SLZ is a potent NF-κβ agent that inhibits folate-dependent enzymes and restricts NF-κβ dependent transcription. It also suppresses phosphorylation of IKβ and nuclear localization of NF-κβ, thus inhibiting cytokines production [18]. According to previous reports, NF-κβ plays a key role in psoriasis [19,20]. This factor regulates a variety of genes involved in different immune as well as inflammatory responses in this disorder [21]. Not only this, it is a key regulator in cellular proliferation, differentiation, and cell apoptosis. Thus, NF-κβ is actively involved in the pathogenesis of this disorder [20]. Keeping in view the above facts, it seems that SLZ can play a promising role in psoriasis. However, there are few studies reported in the literature on its application in this disorder. A report is available suggesting its mechanism as the first-line standard disease-modifying antirheumatic medication treatment for peripheral psoriatic arthritis [22]. To date, no formulation of SLZ has been explored for topical delivery of this moiety in psoriasis. Hence, in light of these facts, the current investigation of SLZ for psoriasis via topical administration is a new and novel strategy. Thus, it has been recommended that the selected prodrug (SLZ) is a good choice in the management of psoriasis [23].

Clinically, SLZ, a class IV moiety with a high log P (3.88), exhibits an absolute bioavailability of up to 15% when administered orally due to its limited solubility and low permeability [24]. The low solubility and permeability of this drug make topical delivery highly challenging despite its low molecular weight (398.39 g/mol). Thus, developing a dual delivery strategy incorporating nanoformulation-engrossed hydrogel may be ideal for the topical delivery of such therapeutic compounds. Meanwhile, no studies have reported on nanodermatology-based topical delivery of SLZ in psoriasis in the literature. In this context, cyclodextrin nanosponges (CD-NS) are promising nanocarriers that are widely utilized in various drug delivery applications to enhance drug solubility, stability, and controlled release [25]. Cyclodextrins are cyclic glucopyranose oligomers, which are prepared by enzyme reaction on hydrolyzed starch. The α, β, and γ are chief native types of cyclodextrins composed of 6, 7, and 8 glucopyranose units, respectively [26]. Herein, we have preferred β-cyclodextrin in the preparation of nanosponges owing to its easy availability, cost-effective nature, augmented drug loading capacity, augmented entrapment efficiency, augmented complexing ability with crosslinking agents, and nano-sized cavities [27]. β-cyclodextrin is cost-effective, poorly water-soluble (1.85% *w*/*v* at 25 °C temperature), and shows toxicity when administered via intravenous route [26]. The fabrication of SLZ nanosponges was carried out using the melt technique, which is solvent-free and helps prevent skin irritation in topical formulations due to the use of toxic solvents. Herein, diphenyl carbonate (DPC) was chosen as a crosslinker as it is environmentally friendly, non-corrosive, and highly suitable for high polycarbonate production [28,29]. Further, the melting point of DPC is 81–82 °C, which is suitable for the melt technique. This innovative delivery system utilizes the unique properties of CDs in developing nanocarriers for sustained and targeted therapy [30]. Numerous studies have demonstrated the significant potential of this carrier for improving drug penetration and therapeutic outcomes in dermatological formulations [31,32,33]. Considering the significance of this carrier, the objective of the current study is to formulate SLZ-loaded NSs and assess their potential for effective therapy in psoriasis. Although this delivery system provides all the merits of nanocarriers, however, owing to their particulate nature, these need to be engrossed with some other topical vehicle like hydrogels for ease of application [34]. In addition, the hydrogels can help in hydrating the skin, and provide an extra advantage in the treatment of psoriasis, where the absence of a natural moisturizing agent is a major issue. The SLZ-loaded NS was prepared by melt polymerization method and examined for various pharmaceutical characteristics. A NS (SLZ-NS4) was chosen to incorporate into hydrogel prepared using Carbopol to facilitate topical application of nanosponges. In vivo anti-psoriatic activity was assessed in a mouse tail and imiquimod (IMQ)-induced mouse model.

## 2. Results and Discussion

Advancements in delivery techniques for therapeutic agents lead to the formation of nanoparticles that have higher drug loading, sustained release, targeted distribution, and prolonged efficacy [35]. A novel formulation comprising SLZ-loaded β-CD nanosponges (SLZ-NS) embedded in Carbopol hydrogel for topical application in psoriasis was developed. After appropriate characterization and in vitro evaluation, the selected batch (SLZ-NS4) was embedded in 1% Carbopol hydrogel and further characterized. Lastly, in vivo evaluation was carried out using the mouse tail model and IMQ-induced inflammation model employing Swiss mice.

The pathogenesis of many diseases, including autoimmune and other inflammatory diseases, is majorly driven by immune reactions [36,37]. These reactions play a pivotal role in psoriasis. However, its interaction with epidermal factors plays a synergistic role in the initiation as well as maintenance of psoriasis. Keeping in mind the complex pathophysiology of psoriasis, the present work was designed. The mouse tail model recapitulates some characteristics of psoriasis. Parakeratosis in the mouse tail is known to be analogous to keratinization in human skin. An innate characteristic of adult mouse tails is known as parakeratosis. Therefore, it is valuable to evaluate the anti-psoriatic efficacy of test samples. In order to evaluate the anti-psoriatic effect of moieties and their topical formulations in the context of the onset of orthokeratosis, the tail model is frequently used as an experimental model for psoriasis [38]. On the other hand, a close resemblance has been observed between human plaque psoriatic lesions and IMQ-induced psoriasis in mice [39]. Researchers commonly use this in vivo model for evaluating antipsoriatic medications [40]. IMQ-induced psoriasis in selected animals was used to further support the findings obtained from the tail model. The significance of targeting and the special qualities of NSs for promoting drug solubilization and release leading to increased drug efficacy has been shown in our earlier study utilizing the NS delivery system [41].

### 2.1. Preparation and Characterization of SLZ-NSs

**Figure 1:**
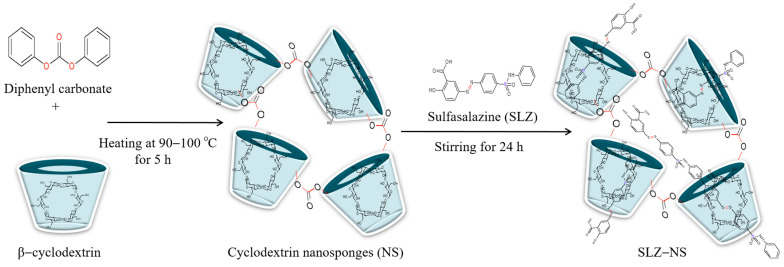
Fabrication of sulfasalazine nanosponges using diphenyl carbonate as a crosslinking agent.

Different batches of SLZ-NSs were fabricated by employing the melt polymerization method with β-CD and diphenyl carbonate. A schematic representation of the fabrication of the SLZ-NSs is illustrated in Figure 1. The first step involves the preparation of placebo NSs with variable molar ratios of DPC and β-CD. In the second step, the SLZ was encapsulated in blank cyclodextrin nanosponges by the lyophilization process. The encapsulation efficiency of the complex between β-CD and SLZ formulations might depend on the method of inclusion complex formation between SLZ and β-CD. The interaction involved between SLZ and β-CD could only occur through the benzoic acid moiety entering from the inner cone of the β-CD [42]. It is also assumed that, alongside the inclusion of the aromatic ring into the macrocyclic cavity, surface interactions take place. Ionized carboxy and amino groups of sulfasalazine are located outside the cavity and interact with OH groups of the CDs [43].

Different physicochemical parameters (solubilization efficiency, mean particle size, zeta potential, PDI, encapsulation, and loading) of SLZ-NSs are listed in Table 1 and Figure S1. From solubilization efficiency findings, the ratio 1:4 (CD/DPC) resulted in NSs with the highest SLZ solubility; this solubility was found to be augmented up to 15 times in comparison to free SLZ (Figure S1). Despite the slight differences in zeta values, −16.93 mV to −11.90 mV, all SLZ-NS batches presented values indicating adequate electrostatic repulsion (repulsive force between particles), avoiding particle aggregation or coalescence [44]. The negative charge of the particles might be due to the ionization of the carboxylic acid group of the SLZ molecule [18]. The PDI values ranged between 0.255 and 0.573, while the size of the particles was between 276 nm and 397 nm. The polydispersity index typically ranges from 0 to 1, with values less than 0.1 indicating monodispersity and values larger than 0.3 indicating heterogeneity [45]. The observed PDI values suggest that the particle distributions in the different SLZ-NS batches exhibit moderate variability in particle sizes. However, the selected batch (SLZ-NS4) had PDI values below 0.3, indicating a narrow particle size distribution. On the basis of solubility, encapsulation efficiency, and loading efficiency, SLZ-NS4 was selected for further evaluation.

Field emission scanning electron microscopy and transmission electron microscopy were utilized to examine the nanoscale architecture of the molecular assemblies. FE-SEM images of SLZ-NSs are illustrated in Figure S2. As depicted here, the obtained NSs showed a porous structure. TEM revealed the morphology of the prepared formulation. As expected, the vesicles appeared smaller in size with homogenous distribution, irregular with a deformed shape, and mostly crystalline (Figure S3). The Fourier transform infrared spectroscopy (FTIR) of samples is displayed in Figure S4. The spectrum of SLZ-NS4 showed SLZ peaks (751, 1029, 1159, 1230, 1774, 1592, 1492, 2926, and 3370 cm^−1^) indicating proper incorporation of SLZ into the NSs. This advocated its encapsulation in nanosponges via inclusion or non-inclusion phenomenon [46,47]. Figure S5 indicates the powder X-ray diffraction (PXRD) of SLZ and SLZ-NSs. Sharp peaks in Figure S5 (11.75, 15.38, 16.89, 18.71, 20.53, 21.74, 23.56, 27.50° 2θ) of SLZ revealed its well-defined crystalline structure [48]. The PXRD pattern of SLZ-NS4 has different crystalline structures (the prominent peaks at 10.53, 12.35, 15.08, 16.89, 19.32, 20.53, 22.44, and 26.84° 2θ) compared with its original constituents. Thermal properties of SLZ and SLZ-NS4 were determined using differential scanning calorimetry (DSC) and are represented in Figure S6. SLZ displayed an endothermic point at 262.22 °C (enthalpy: 165.88 J/g) related to the melting point of the drug followed by an exotherm corresponding to its decomposition at higher temperatures (286.54 °C; enthalpy: 243.96 J/g) (Figure S6). SLZ-NS4s did not show the endotherm of SLZ, which again confirmed SLZ had been successfully included in NSs, and also, there could be a tight interaction between CD and drug molecules, as described in the literature [49,50,51]. In order to assess the modification in chemical as well as physical features of nanosponges, the thermal behavior of SLZ and SLZ-NS4 was checked (Figure S7). The results of thermogravimetric analysis (TGA) demonstrated the produced nanoformulation’s thermal stability and reinforced the DSC findings.

### 2.2. In Vitro Release of SLZ

**Figure 2:**
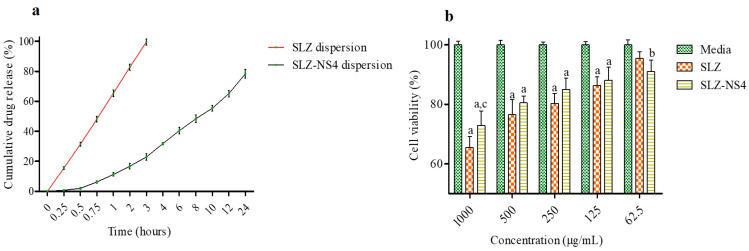
(**a**) The SLZ release curves from the SLZ dispersion and SLZ-NS4 dispersion within 24 h. (**b**) Effect of SLZ and SLZ-NS4 on THP1 cells (24 h). Data given here are the average of three trials. Statistics by two-way ANOVA and Bonferroni post-tests. (a) *p* < 0.001 vs. media at particular concentrations, (b) *p* < 0.01 vs. media at particular concentrations and (c) *p* < 0.05 vs. SLZ at 1000 µg/mL concentration. SLZ: Sulfasalazine; SLZ-NS4: Sulfasalazine-loaded nanosponges.

As represented in Figure 2a, SLZ dispersion demonstrated 99.57 ± 2.00% release in 3 h with 65.47 ± 2.20% release in 1 h; however, SLZ release from the NSs was comparatively slow (22.98 ± 2.24%) in 3 h and 1 h (11.31 ± 1.22%) of study. In addition to superior physicochemical parameters, SLZ-NS displayed a slow release up to 24 h (78.34 ± 2.88%) (Figure 2a). This slow release may be accredited to the inclusion of SLZ into cavities as well as the crosslinked structure of nanosponges; this also confirmed the successful complexation of SLZ and NSs. Various research teams have reported similar data with low release of encapsulated compounds from NSs [50,52]. According to a different study, topical administration of NSs has advantages over conventional formulations in terms of sustained release properties [47,53,54]. By avoiding its frequent dermal application, the delayed release of SLZ from nanoformulations enhances its safety feature in the treatment of psoriasis. Additionally, the Higuchi model’s in vitro drug release from SLZ-NS4 showed the greatest r^2^ value (0.975), confirming SLZ release by diffusion (Table 2). Indeed, the Higuchi equation has been employed for diffusion-controlled release from a porous matrix where the bioactive molecule is released by the bathing release medium that enters the nanocomplex matrix via capillaries and pores [47,55,56].

### 2.3. Cytocompatibility Assay of SLZ-NS4 Using THP1 Cell Line

In vitro cytotoxicity of SLZ and SLZ-NS4 was investigated against THP1 (human monocytes) cells, and their viability was assessed using an MTT assay (Figure 2b). The concentrations (62.5, 125, 500, and 1000 μg/mL) of SLZ-NS4 had a remarkable effect (*p* < 0.01) on cell viability. The findings revealed that toxicity of SLZ-NS4 was found to be concentration-dependent, although on increasing the concentration of nanoformulation up to 1000 μg/mL, cell viability was found to be reduced. Higher concentrations showed a remarkable (*p* < 0.001) influence on the viability of THP1 cells (Figure 2b). The findings of this study indicated that the entrapment of SLZ into the NS augmented its cell viability by decreasing the direct interaction of the drug with the cell membrane. Herein, the outcomes may be accredited to the slow release of SLZ from SLZ-NS4, advocating the safety of the fabricated nanoformulation. Similar findings were obtained for dithranol nanosponges examined on the THP1 cell line by our research group [41]. Recently, Heikal and the research team investigated the cytotoxicity of baicalin rhamno-nanocapsules with THP1 cells [57].

### 2.4. SLZ-NS Embedded Hydrogel Fabrication and Its Characterization

**Figure 3:**
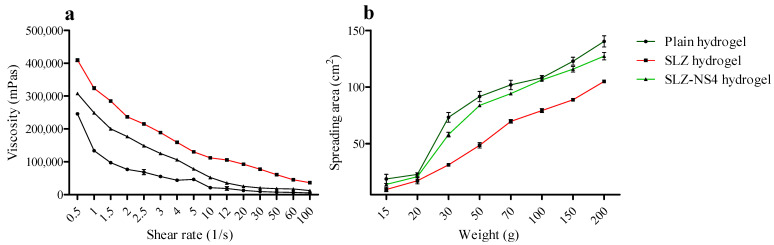
Assessment of rheology-prepared gels. (**a**) Shear rate vs. viscosity of hydrogels; (**b**) Spreadability of hydrogel samples. Data given here are the average of three trials.

CD-based NSs are solid hypercrosslinked polymer structures. Nanosponges’ solid powder cannot be applied directly to the skin due to its particulate nature. Thus, Carbopol 934-based hydrogel was selected as a vehicle, as it is widely used in skin formulations [58,59,60]. The selected formulation (SLZ-NS4) was amalgamated into a hydrogel formulation employing 1% Carbopol 934 as a gelling agent, based on the literature [61]. Both SLZ hydrogel (SLZ-HG) and SLZ-NS4 hydrogel (SLZ-NS-HG) were found to be homogenous and smooth. The hydrogels possess a semisolid consistency and are convenient to spread on the skin surface. The pH of the fabricated SLZ-NS-HG was observed to be from 5.4 to 5.6. The graph of shear rate vs. measured viscosity of each prepared hydrogel revealed that a reduction in viscosity occurs with an increase in shear rate (Figure 3a), suggesting the pseudoplastic (shear thinning) behavior of prepared hydrogels [62]. Further, the viscosity of the developed nanogel was found to be reduced and almost similar to the plain Carbopol hydrogel upon entrapment of SLZ-NS4, as expected due to the nanosize of the amalgamated carriers [63]. Rheograms showing the shearing behavior of long-chain Carbopol molecules have been reported elsewhere [64,65].

The initial spreading area of plain HG and SLZ-NS-HG was noted as 18.85 ± 4.06 and 13.98 ± 1.19 cm^2^, respectively; on the other hand, SLZ-HG showed an initial spreading area of 9.59 ± 2.03 cm^2^. On applying 200 g weight, the spreading area for plain HG, SLZ-NS-HG, and SLZ-HG were recorded as 140.36 ± 4.94, 127.35 ± 3.29 and 104.98 ± 1.00 cm^2^, respectively (Figure 3b).

The results of texture analysis compiled in Table 3 demonstrated excellent gel strength to preserve its structure during storage, good spreadability, and could easily be extruded from a tube, all of which make it a good choice for topical application [66]. Additionally, the hydrogel systems have demonstrated sufficient cohesiveness or good bioadhesion, which would enable their prolonged availability on the application site [66]. The prepared hydrogels are fit for topical administration because of these properties.

### 2.5. Safety Assessment and Mechanistic Investigation of SLZ-NS Hydrogel

**Figure 4:**
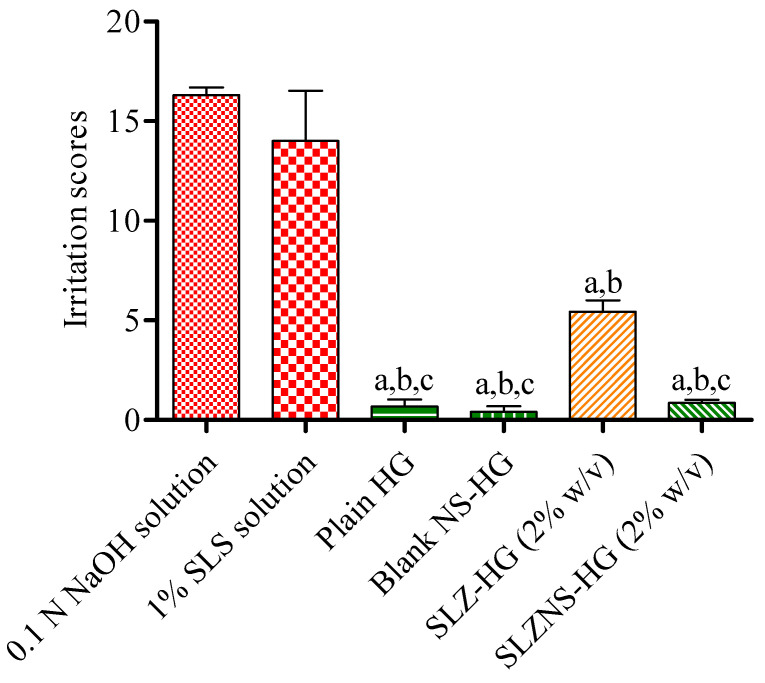
Irritation findings for test samples (average of 3 eggs). Statistics by one-way ANOVA and Tukey’s test. (a) *p* < 0.001 (in comparison to 0.1 N sodium hydroxide solution), (b) *p* < 0.001 (in comparison to 1% sodium lauryl sulfate solution), (c) *p* < 0.01 (in comparison to SLZ-HG). SLZ-HG: Sulfasalazine hydrogel; SLZ-NS-HG: Sulfasalazine nanosponge (NS) hydrogel; Blank NS-HG: Placebo NS hydrogel; and Plain HG: Plain hydrogel.

SLZ-NS hydrogels were evaluated for irritation behavior in a comparison with SLZ-HG employing the Hen’s Egg Test Chorioallantoic Membrane (HET-CAM) study. This approach serves as a notable tool to assess irritation of various formulations, such as particles, gels, and emulsions [67]. Outcomes of irritation assessment on CAM are consolidated and presented as irritation score (IRSc) in Figure 4. The CAM did not exhibit any irritation response to either Carbopol hydrogel (1% *w*/*v*; IRSc: 0.68 ± 0.35) or blank NS hydrogel (IRSc: 0.4067 ± 0.28). The calculated irritation score for SLZ hydrogel was 5.44 ± 0.56, categorizing it as a moderate irritant (score range of 5.0–8.9), and this was significant statically (*p* < 0.001) when compared to positive control. However, when SLZ-NS-HG (equal to SLZ 2% *w*/*v*) was applied, no adverse reactions were observed, demonstrating the safer nature of the developed nano hydrogel (IRSc: 0.86 ± 0.15, non-irritant) (Figure 4). Based on the results obtained from this assay, it is evident that the SLZ-NS-HG formulation does not induce any irritation, advocating its safety on psoriatic skin. Recently, similar studies have been carried out for tacrolimus-loaded polymeric nanocapsules amalgamated with pectin-based hydrogel to ensure safe cutaneous delivery for psoriasis [68].

### 2.6. Assessment of Depth of Skin Permeation of SLZ-NS-HG

**Figure 5:**
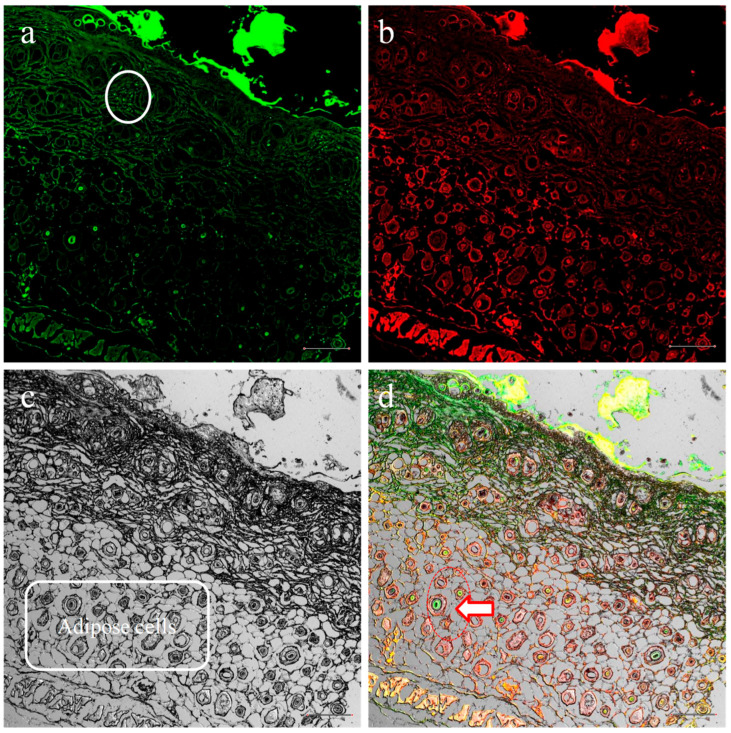
FITC-SLZ nanosponge-based hydrogel treatment (24 h) together with confocal laser scanning microscope images. Images of (**a**) green fluorescence filters, (**b**) red fluorescence filters with skin structure labels, (**c**) phase contrast filters, and (**d**) overlays of (**a**,**b**). FITC-SLZ-laden nanosponges in the dermis are represented by dot-shaped white circles. The skin’s dermis has the highest concentration of green fluorescence, as shown in (**d**). Adipose cell dye deposition is indicated by red/white arrow in three dimensions. Original magnification is ×100. The scale bar in all images is 1000 µm long.

Psoriasis primarily starts in the epidermal layer of the skin, where the abnormal cell growth disrupts the integrity of the basement membrane. This results in the invasion of the dermis by compact masses or layers of tumorous cells in the epidermis. Therefore, to optimize treatment outcomes, it is crucial for the medication to be delivered precisely between the epidermal and dermal layers of the skin [69]. Confocal laser scanning microscopy experiments were carried out to assess the cellular uptake patterns and the penetration of fluorescein isothiocyanate (FITC)-SLZ-loaded NS hydrogel. To evaluate the actual carrier function, distribution, and penetration depth of the developed gel, FITC fluorescent dye was examined in the skin layers 24 h after topical application of the formulation. The outcomes of the dye-loaded SLZ-NS-HG are displayed in Figure 5, where FITC demonstrated higher florescence intensity, viable epidermis, and increased penetration depth into the (SC) layer. Figure 5a,d reveals evident permeation via intracellular channels of the SC. Similar results were reported in another study, as well [70]. The permeability barriers were locally decreased by the highly stained structures acting as permeability shunts. Prominently fluorescent spherical structures observed in annexes of skin supported transport of the dye encapsulated in nanocarriers (Figure 5a). The improved dye retention in the skin’s deeper layers might have been facilitated by the hydrogel base. Results were positive and supported the improved efficacy of SLZ after encapsulation in the nanocarrier followed by integration in the hydrogel.

### 2.7. In Vivo Anti-Psoriatic Studies

#### 2.7.1. Mouse Tail Model

##### Histopathological Assessment

**Figure 6:**
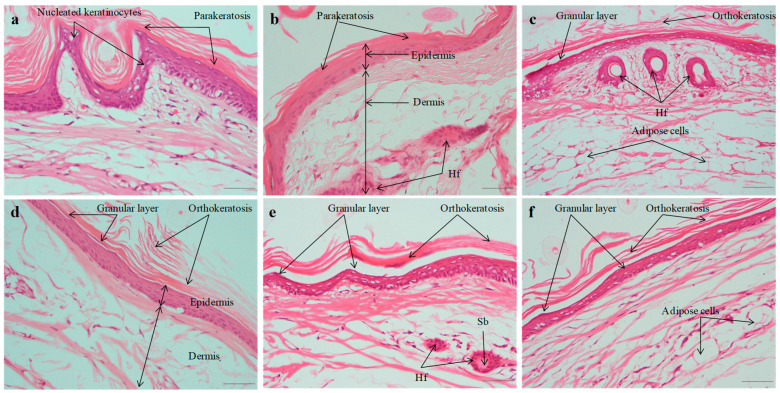
Histopathology of mouse tail skin after topical treatment with (**a**) Control (no treatment), (**b**) SLZ-HG 1% *w*/*v*, (**c**) SLZ-HG 2% *w*/*v*, (**d**) Compound dithranol ointment, (**e**) SLZ-NS-HG 1% *w*/*v* equivalent to SLZ, (**f**) SLZ-NS-HG 2% *w*/*v* equivalent to SLZ for 14 days. Original magnification is ×400. The scale bar in all images is 100 µm long. Hf: Hair follicle, Sg: Sebaceous gland.

Histological characteristics of stained tail segments (previously treated with different test specimens) are shown in Figure 6. There were clear signs of parakeratosis (a higher density of nucleated keratinocytes) and a thinning or absence of granular layer from the sham group (Group 1). Tail skin sections treated for fourteen days with SLZ-NS-HG showed significant histological differences compared to traditional SLZ-HGs. During this time span, ortho-keratotic SC provinces expanded longitudinally in the preceding parakeratotic state. Drug activity, relative epidermal thickness, and orthokeratotic scores varied across all groups (Table 4).

The outcomes indicated that SLZ-NS-HG had a higher anti-psoriatic effect (*p* < 0.001) with respect to plain and SLZ-HG (Table 4). The possible reduction in the dose of SLZ with respect to pure form may be attributed to the entrapment of SLZ in CD nanocarriers, which facilitates its anti-psoriatic action. Furthermore, the increased activity of SLZ-NS-HGs could be attributed to the diffusion of NSs loaded with SLZ and targeting of the skin epidermal layer. The current findings are consistent with the improved anti-psoriatic efficacy observed upon topical application of babchi oil NS hydrogel and clobetasol propionate NS hydrogel [71,72]. When compared to SLZ conventional gel, the produced nano hydrogel increased the anti-psoriatic potential of the drug, ostensibly because the SLZ nanocarrier interacted with the skin strata more effectively.

##### Anti-Psoriatic Enzymes and Oxidative Stress Estimation

Since psoriasis has been associated with the growth of keratinocytes, oxidative stress is crucial for its pathophysiology. Enzymatic antioxidants (which act as a defense pathway), such as reduced glutathione (GSH), superoxide dismutase (SOD), reductase, catalase, and thioredoxin reductase are found in the skin [73]. Oxidative stress contributes to ROS imbalance, reducing the levels of antioxidant enzymes, which causes molecular abnormalities and redox-signaling disintegration, as documented in the literature. The subsequent redox imbalance plays a crucial part in the development of psoriasis [74]. Thus, in the current study, the effects of gel-loaded SLZ-NSs on oxidative stress markers were examined employing a mouse tail model. The beneficial effects of this nanogel in reducing inflammation in the skin have been hypothesized in light of the previously mentioned role of oxidative stress in this skin disorder and prodrug SLZ in minimizing inflammation as well as oxidative damage. While the precise mechanism of action of SLZ and its metabolites remains incompletely elucidated, the antioxidant effects of this moiety are likely attributable to its ability to scavenge reactive oxygen (ROS) as well as nitrogen species (RNS). This ability may play a remarkable role in the anti-inflammatory effects of SLZ [75]. It has been shown previously that diminished antioxidant levels in mouse tail skin homogenate and increased ROS generation, followed by epidermal keratinocyte proliferation, are the major factors responsible for contributing to psoriasis [72].

**Figure 7:**
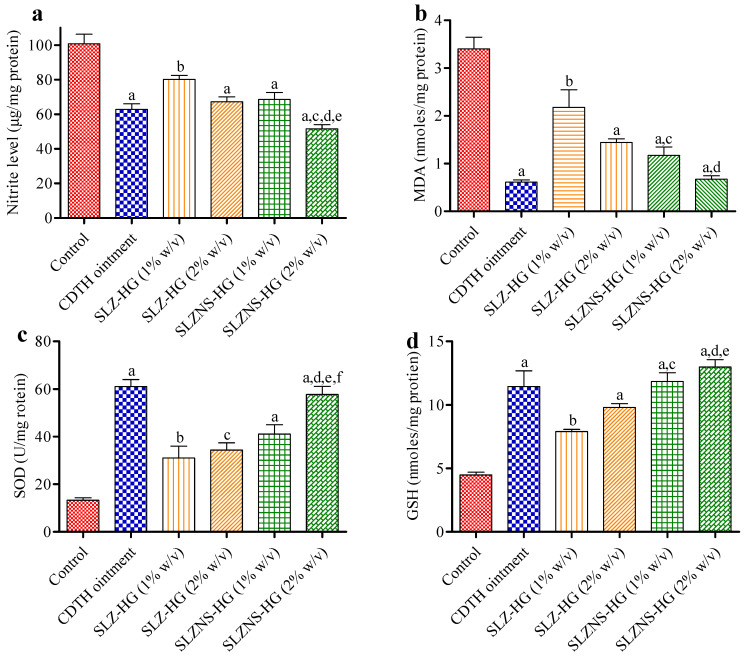
Results of (**a**) Nitric oxide (NO) expression, (**b**) Malondialdehyde (MDA) levels (**c**) Superoxide dismutase (SOD) levels, and (**d**) Reduced glutathione (GSH) levels. Data given here are the average of six trials. Statistics by one-way ANOVA and Tukey’s test. CDTH ointment: Compound dithranol ointment, SLZ-HG: Sulfasalazine-loaded hydrogel, SLZ-NS-HG: Sulfasalazine nanosponge-loaded hydrogel. Figure (**a**): (a) *p* < 0.001 versus control, (b) *p* < 0.01 versus control, (c) *p* < 0.001 versus SLZ-HG 1% *w*/*v*, (d) *p* < 0.05 versus SLZ-HG 2% *w*/*v*, (e) *p* < 0.05 versus SLZ-HG 1% *w*/*v.* Figure (**b**): (a) *p* < 0.001 versus control, (b) *p* < 0.01 versus control, (c) *p* < 0.05 versus SLZ-HG 1% *w*/*v*, (d) *p* < 0.001 versus SLZ-HG 2% *w*/*v*. Figure (**c**): (a) *p* < 0.001 versus control, (b) *p* < 0.01 versus control, (c) *p* < 0.05 versus control, (d) *p* < 0.001 versus SLZ-HG 1% *w*/*v*, (e) *p* < 0.001 versus SLZ-HG 2% *w*/*v,* (f) *p* < 0.05 versus SLZ-HG 1% *w*/*v*. Figure (**d**): (a) *p* < 0.001 versus control, (b) *p* < 0.01 versus control, (c) *p* < 0.01 versus SLZ-HG 1% *w*/*v*, (d) *p* < 0.001 versus SLZ-HG 1% *w*/*v*, (e) *p* < 0.05 versus SLZ-HG 2% *w*/*v*.

In the current research, levels of NO (nitrite) were observed as control group (Plain-HG) ˃ SLZ-HG (1% *w*/*v*) ˃ SLZ-NS-HG (equivalent to SLZ 1% *w*/*v*) ˃ SLZ-HG (2% *w*/*v*) ˃ compound dithranol ointment ˃ SLZ-NS-HG (equivalent to SLZ 2% *w*/*v*) (Figure 7a). Additionally, NO levels were significantly lower in the SLZ-NS-HG (equivalent to SLZ 2% *w*/*v*) group (*p* < 0.05), with respect to SLZ-HG (2% *w*/*v*) (Figure 7a), strongly supporting its superior pharmacological efficacy. These results align with histopathological observations, providing corroborating evidence for the enhanced therapeutic action of the fabricated nanoformulation-based hydrogel of SLZ.

Likewise, a notable quantity of MDA (malondialdehyde) was noted in the Swiss mice’s untreated tail (control group). MDA content was significantly low (*p* < 0.01) in the topical application of SLZ hydrogel (1 and 2% *w*/*v*), SLZ-NS-HG (equal to SLZ 1 and 2% *w*/*v*), and positive control group than in the control group (Figure 7b). However, this reduction was significantly higher in nanoformulations than the plain SLZ-HG (1% *w*/*v*), highlighting their efficacy in psoriasis. The current results substantiated the decreased SOD amount in control animals (psoriatic skin). However, with topical administration of SLZ-NS-HG (equivalent to SLZ 2% *w*/*v*), SOD content was observed to be significantly (*p* < 0.001) higher in treated animals in relation to the control group and the plain SLZ-HG (1% and 2% *w*/*v*) (Figure 7c). These observations align with the trends observed in NO levels. On topical application of SLZ-NS-HG (equivalent to SLZ 2% *w*/*v*), GSH levels were observed to be restored and increased (*p* < 0.01) when compared to the control group and SLZ-HG (1% *w*/*v*) (Figure 7d). This action might have led to the mitigation of excessive ROS through neutralization. The novel finding was that topical administration of SLZ-NS4 gel ameliorated oxidative stress and lipid peroxidation in this formulation. The advantages of fabricated SLZ-loaded NS hydrogel over plain SLZ hydrogel for regulating ROS production and associated consequences in psoriatic skin have been established based on biochemical estimation results (NO, SOD, GSH, and MDA levels). Reduced oxidative stress and an evident onset of orthokeratosis were demonstrated by these indicators.

#### 2.7.2. IMQ-Induced Psoriasis Mouse Model

**Figure 8:**
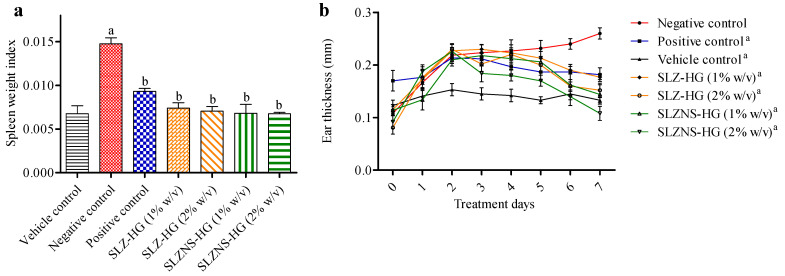
Reduction in splenomegaly (**a**) and left ear thickness (**b**) in an in vivo psoriatic mice model after anti-psoriatic nanoformulation treatments. Data given here are the average of six trials. Statistics by one-way ANOVA and Tukey’s test for spleen weight index. Figure (**a**): (a) *p* < 0.001 in comparison to vehicle control, and (b) *p* < 0.001 in comparison to negative control. Statistics by one-way ANOVA and Bonferroni post-tests for ear thickness. Figure (**b**): (a) *p* < 0.001 (compared to the negative control at the end of the treatment therapy) were used to examine the data after a two-way ANOVA. SLZ-HG: Sulfasalazine hydrogel; SLZ-NS-HG: Sulfasalazine nanosponge hydrogel; Negative control: Imiquimod-treated; Vehicle control: Plain hydrogel-treated only; Positive control: Marketed ointment (Derobin)-treated.

**Figure 9:**
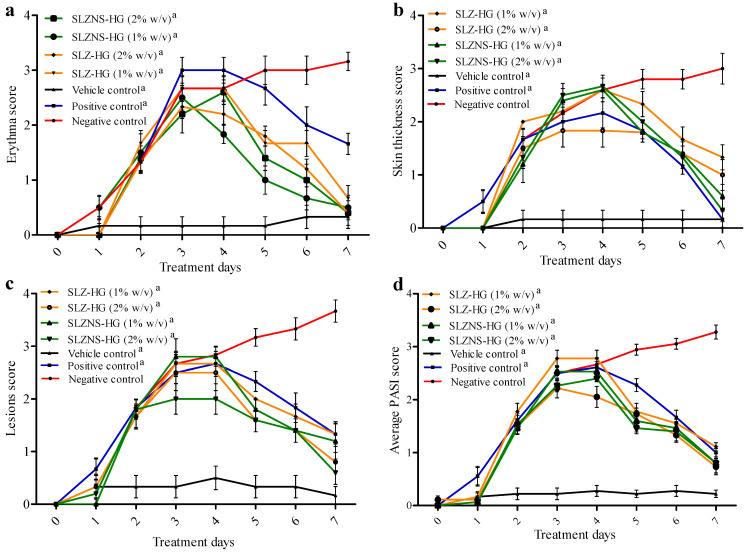
Phenotypical changes in the psoriatic dorsal portion of Swiss mice in various groups (*n* = 6) were assessed for one week, such as erythema (**a**), skin thickness (**b**), lesions score (**c**), and average PASI score (**d**) of the dorsal skin, with a scale from 0 to 4. Data given here are the average of six trials. Statistics by one-way ANOVA and Bonferroni post-tests. (a) *p* < 0.001 vs. negative control at the end of the treatment period. SLZ-HG: Sulfasalazine hydrogel; SLZ-NS-HG: Sulfasalazine nanosponge hydrogel; Negative control: Imiquimod-treated only; Vehicle control: Plain hydrogel-treated only; Positive control: marketed ointment (Derobin)-treated. The groups (except vehicle control) were treated with IMQ cream, and after 4 h, the test formulation was applied topically.

Swiss mice’s dorsal side showed a variety of phenotypic changes after receiving topical administration of IMQ cream (5% *w*/*w*) for seven continuous days. These alterations comprised erythema, redness, and white, silvery scales, each of which was visually identified and scored independently (Figure 8 and Figure 9). The degree of inflammatory lesions was assessed as these changes progressed [39]. To further clarify the effect of the SLZ novel formulation in psoriasis management, a psoriasis-like skin lesions mouse model induced by IMQ was utilized. As represented in Figure 8, spleen–bodyweight index (SBWI), ear thickness, erythema, skin thickness, scaling, and cumulative psoriasis area and severity index (PASI) score were significantly augmented in IMQ-induced Swiss mice (negative control) with respect to that in control mice. A loss in body weight of animals treated with IMQ indicates their poor health condition [76]. Notably, SLZ formulations (SLZ-HG 1%, SLZ-HG 2%, SLZ-NS-HG 1%, SLZ-NS-HG 2%) and positive control (Derobin ointment) could remarkably decline the cumulative PASI score, ear thickness, erythema, skin thickness, scaling, and SBWI in affected lesions of IMQ-induced mice (Figure 8 and Figure 9). On treatment with marketed ointment (dithranol 1.15% *w*/*w*), normalization of these symptoms was observed, which may be because of its well-known high potency in treating psoriasis. SLZ formulations alleviated the IMQ-induced mouse skin lesions and decreased the PASI score in the affected areas. Furthermore, at both test dose levels, the results of SLZ-NS-HG treatment were similar to those of plain SLZ-HG. The encouraging outcomes with SLZ-NS-HG (corresponding to 1 and 2% *w*/*v* of SLZ) demonstrated the potential of the produced nanoformulation in treating psoriasis by confirming the effectiveness of SLZ entrapment in NSs and their hydrogel integration.

In the case of SLZ conventional hydrogel-treated mice (SLZ 1% *w*/*v*), the reduction in PASI was 66.66 ± 3.33, representing its poor antipsoriatic effect (Table 5), whereas a reduction in PASI was observed at 77.18 ± 5.02 for SLZ hydrogel (2% *w*/*v*). In the case of SLZ-NS-HG (equivalent to SLZ 1 and 2% *w*/*v*), the decline in the PASI score was found to be 81.68 ± 3.61 and 84.86 ± 5.74, respectively, confirming its effectiveness in psoriasis, as mentioned earlier [76].

#### 2.7.3. Histopathological Evaluation

**Figure 10:**
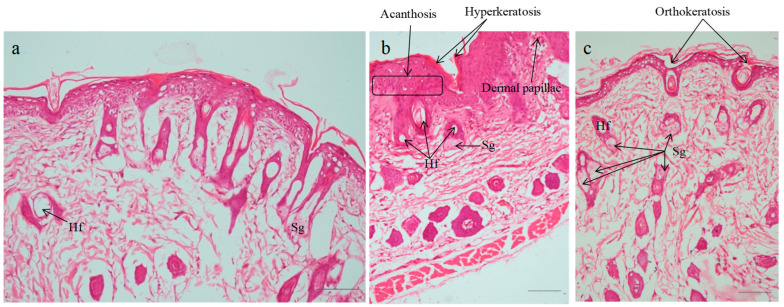
Histopathology assessments in mice for induction of psoriasis and marketed cream. Images of back skin slices of (**a**) Control, (**b**) Negative control, and (**c**) Positive control. H.&E., original magnification is ×200. Hf: Hair follicle, Sg: Sebaceous gland.

**Figure 11:**
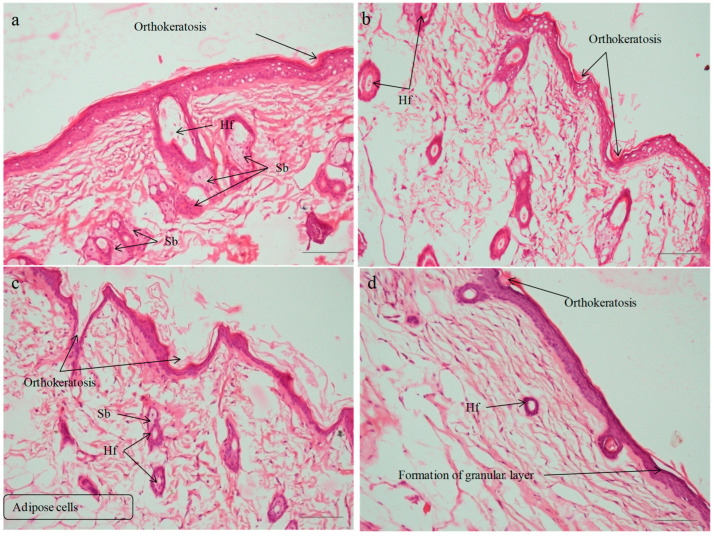
Histopathology assessments for sulfasalazine nanosponge hydrogel. Images of skin sections (back) of mice from various groups; (**a**) SLZ hydrogel (1% *w*/*v*), (**b**) SLZ hydrogel (2% *w*/*v*), (**c**) SLZ-NS hydrogel (equivalent to SLZ 1% *w*/*v*) and (**d**) SLZ-NS hydrogel (equivalent to SLZ 2% *w*/*v*). H.&E., original magnification is ×200 (Hf: Hair follicle, Sb: Sebaceous gland).

The histological alterations in the skin caused by IMQ cream and subsequent treatment with various formulations were observed through the use of hematoxylin–eosin (HE) staining on the dorsal skin. Figure 10 shows images of skin from various groups. Histology of normal skin was seen in the vehicle control group that was treated with plain hydrogel (Figure 10a). Histopathology of negative control (IMQ-treated group) is characterized by hyperkeratosis, parakeratosis, acanthosis, and epidermal infiltrates in the dermis layer. In the negative control (IMQ-treated animals; Figure 10b), histological evaluations showed a ~4-fold (3.85) boost in epidermal thickness of SC, characterized via hyperproliferation of keratinocyte cells, acanthosis with elongated rete-like ridges, as well as perivascular infiltration of inflammatory cells in the upper dermis. This phenotype closely resembled that seen in human psoriatic skin [39,77]. Findings were found corroborating previous studies. Furthermore, a notable hyperkeratosis along with aberrant keratinocyte differentiation was noted. Epidermal thickening was caused by basal and suprabasal keratinocyte hyperplasia. The positive control group (Derobin ointment) exhibited a decline in hyperkeratosis and acanthosis with more regular epidermis observed, but it was thicker than that of the normal group (Figure 10c). The induced psoriasis was markedly reduced by treatment with SLZ-HG (1 and 2% *w*/*v*) and SLZ-NS-HG (equivalent to SLZ 1 and 2% *w*/*v*) (Figure 11 a–d). The promising results exhibited herein may be due to the immunosuppressive, anti-inflammatory, and anti-bacterial effects of SLZ, as reported earlier [16,18].

The anti-psoriatic potential of SLZ-NS-HG (equivalent to SLZ 1% *w*/*v*) was found to be equivalent to that of SLZ-HG (2% *w*/*v*). The reduction in epidermal thickening was less pronounced for both SLZ-HG (1 and 2% *w*/*v*) than SLZ-NS-HGs. These findings suggested that by lowering the dosage of SLZ and addressing its physicochemical issues, SLZ-NS-HG may act as an effective nanocarrier in the management of psoriasis. This effect was supported by the increased retention of SLZ-loaded NSs in skin layers and their deeper penetration into the skin. The histopathological findings were in line with the PASI assessment. Owing to efficient encapsulating, enhanced solubilization, and localizing at the application site, SLZ-NS-HG was intended to decrease the total dose and consumption of SLZ based on its antipsoriatic activity. This would not only maximize the therapeutic effect but minimize the systemic side effects.

### 2.8. Oxidative Stress and Antioxidant Enzyme Estimation

**Figure 12:**
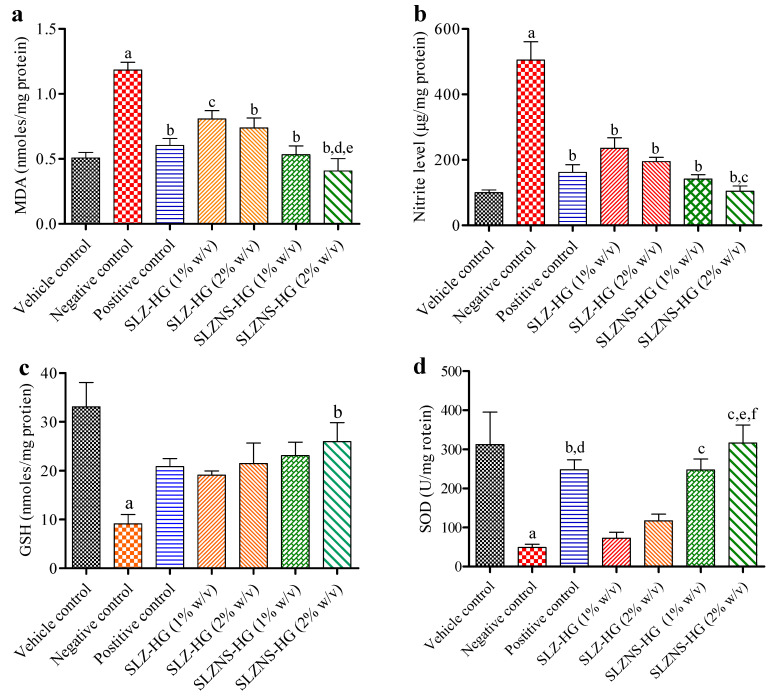
Effect of SLZ and its nanoformulation on skin tissue oxidative stress markers. The decreased (**a**) MDA and (**b**) NO levels while elevated (**c**) GSH and (**d**) SOD levels in skin tissue (back) of various experimental mice. Data given here are the average of six trials. Statistics by one-way ANOVA and Tukey’s test. Sulfasalazine-containing hydrogel (SLZ-HG); sulfasalazine nanosponge hydrogel (SLZ-NS-HG); negative control: group treated with imiquimod; marketed ointment as a positive control. For Figure (**a**): (a) *p* < 0.001 versus vehicle control, (b) *p* < 0.001 versus negative control, (c) *p* < 0.01 versus negative control, (d) *p* < 0.01 versus SLZ-HG 1% *w*/*v*, and (e) *p* < 0.05 versus SLZ-HG 2% *w*/*v*. For Figure (**b**): (a) *p* < 0.001 versus vehicle control, (b) *p* < 0.001 versus negative control, and (c) *p* < 0.05 versus SLZ-HG 1% *w*/*v*. For Figure (**c**): (a) *p* < 0.001 compared to vehicle control, (b) *p* < 0.05 compared to negative control. For Figure (**d**): (a) *p* < 0.001 versus vehicle control, (b) *p* < 0.05 versus negative control, (c) *p* < 0.001 versus negative control, (d) *p* < 0.05 versus SLZ-HG 1% *w*/*v*, (e) *p* < 0.01 versus SLZ-HG 1% *w*/*v*, (f) *p* < 0.05 versus SLZ-HG 2% *w*/*v*.

To support the findings of the tail model, this experimental work is designed to evaluate the impacts of ROS, antioxidant status, and subsequent effects on oxidative stress indicators in the IMQ-induced psoriasis model after SLZ and its nanogel were applied. Herein, MDA levels were found to be significantly (*p* < 0.001) elevated in IMQ cream-treated mice (negative control) with respect to the vehicle control group, suggesting that psoriasis had been successfully induced in the Swiss mice. Topical administration of fabricated SLZ-NS-HG (equivalent to SLZ 1 and 2% *w*/*v*), positive control, and SLZ-HG (1 and 2% *w*/*v*) produced a statistically significant (*p* < 0.01) decrease in MDA with respect to the negative control group (IMQ treated mice) (Figure 12a). Furthermore, MDA levels for SLZ-NS-HG (equal to SLZ 2% *w*/*v*) were estimated to be decreased (*p* < 0.01 and *p* < 0.05) when compared to SLZ-HG (1 and 2% *w*/*v*, respectively).

NO levels were considerably higher in the negative group after applying IMQ cream (*p* < 0.001) compared to the vehicle group (Figure 12b). This is because the keratinocyte proliferation was facilitated by increased oxidative stress via the NO signaling pathway, as mentioned in the literature [78,79]. NO levels, which were elevated following IMQ cream application, were significantly (*p* < 0.001) decreased by the standard (positive control), SLZ-NS-HG (equal to SLZ 1 and 2% *w*/*v*), and SLZ-HG (SLZ 1 and 2% *w*/*v*) formulations (Figure 12b). Moreover, NO levels were noted to be remarkably lower (*p* < 0.05) in the SLZ-NS-HG-treated group (equivalent to SLZ 2% *w*/*v*) than in the SLZ-HG (1% *w*/*v*) group. Further, these observations were supported by histopathological results, providing additional proof of the enhanced efficacy of SLZ-NS-HG (equivalent to SLZ 2% *w*/*v*). For this type of skin condition, SLZ-NS-HG, which is equivalent to SLZ 2% *w*/*v*, might be a promising option.

When IMQ cream was applied to mice skin, GSH levels were significantly decreased (*p* < 0.001) in comparison to the control group. When marketed ointments, SLZ-HG (1 and 2% *w*/*v*), and SLZ-NS-HG (equivalent to SLZ 1% *w*/*v*) were applied, GSH levels increased. However, in comparison to the negative group (IMQ-treated) (Figure 12c), there was a significant (*p* < 0.05) increase in the case of SLZ-NS-HG (equivalent to SLZ 2% *w*/*v*). These results suggested a reduction in oxidative stress, as mentioned earlier.

After applying IMQ cream, a significant (*p* < 0.001) drop in SOD content was noted in the negative group. Topical application of test samples in conjunction with SLZ-NS-HG (equivalent to SLZ 1% *w*/*v*) and standard (positive control) significantly (*p* < 0.05) increased SOD levels, supporting their potential in reducing oxidative stress. Furthermore, SOD levels were significantly elevated (*p* < 0.001) in the SLZ-NS-HG-treated mice (equivalent to SLZ 2% *w*/*v*) when compared to negative control in this investigation (Figure 12d). Further, SOD contents for SLZ-NS-HG (equal to SLZ 2% *w*/*v*) were noted to have remarkably decreased (*p* < 0.01 and *p* < 0.05) as compared to SLZ-HG (1 and 2% *w*/*v*, respectively). The outcomes of this investigation further substantiate the association between oxidative stress and psoriasis; i.e., when skin oxidative stress was attenuated with SLZ novel gel in psoriatic skin, the skin functions improved. Our results confirmed SLZ’s effectiveness in improving IMQ-induced psoriasis in Swiss mice and opened a new pathway for this skin disorder in clinical practice. When the results of this investigation are considered collectively, it can be concluded that NS-based hydrogel is a promising treatment option for psoriasis.

SLZ-NS-based hydrogel has been extensively investigated in the current study, employing in vitro and in vivo experiments. Additionally, characterization of nanosponges as well as nanosponge-based hydrogel for moiety, irritation assay, and CLSM studies have been carried out. However, the limitation of this work includes in-depth mechanistic investigation. Detailed studies involving the investigation of formulation for the action of NF-κβ in inhibiting cytokine production, cellular proliferation, and cell apoptosis can be assessed in future research studies.

## 3. Materials and Methods

### 3.1. Materials

SLZ was graciously supplied by Ind-Swift Laboratories (Derabassi, India). β-CD was supplied ex gratis by Roquette India (Viramgam, India). DPC and Carbopol 934 were purchased from Sigma Aldrich (Milan, Italy) and Loba Chemie (Mumbai, India), respectively. Sodium chloride and Triethanolamine were obtained from SD Fine Chem (Mumbai, India). Dialysis membrane (molecular weight cut-off: 12,000–14,000 Da) and Flourosein isothiocyanate isomer I were procured from Hi-media Labs (Mumbai, India). THP1 cell lines were obtained from the National Centre for Cell Science (Pune, India). Cell culture reagents (Cat No-10270106) were obtained from Gibco/Invitrogen (Life Technologies) (Paisley, UK). Antibiotic–antimycotic 100X solution (Cat No-15240062) was procured from Thermo Fisher Scientific (Bengaluru, India). Sodium lauryl sulfate was procured from Central Drug House (*p*) Ltd. (New Delhi, India). Derobin ointment [Dithranol, (1.15% *w*/*w*), coal tar (5.3% *w*/*w*), and salicylic acid (1.15% *w*/*w*)] and IMQ cream (5% *w*/*w*) was obtained from USV Pvt. Ltd. (Mumbai, India) and Glenmark Pharmaceuticals (Goa, India), respectively.

### 3.2. Animals and Ethical Compliance

The current research was strictly conducted in adherence to the prescribed standards governing the care, treatment, and utilization of laboratory animals. All animal experimentation was conducted in accordance with the approved protocol sanctioned by the Institutional Animal Ethics Committee of Guru Jambheshwar University of Science and Technology, Hisar, Haryana, India, to supervise and regulate the experiments involving Swiss albino mice. For the current investigation, adult male mice (weight 20–25 g) were selected. Prior to the commencement of the experiments, the mice were allowed to acclimatize to the laboratory environment for a time span of 10 days. During the study, mice were kept in a controlled environment (25 °C ± 1 °C and a 12-h light/dark cycle). They were also given unlimited access to conventional animal food and water.

### 3.3. Analysis of Drugs

SLZ was analyzed in DMSO and phosphate buffer (pH 5.4) using a UV spectrophotometer (Varian Cary-5000, Christ, The Netherlands). Samples in DMSO and phosphate buffer (pH 5.4) were measured at λ_max_ = 360 nm in the concentration range of 1–6 μg/mL. The details of validation are presented in Table S1.

### 3.4. Preparation and Characterization of SLZ-NS

CD-based NSs were synthesized by the melt polymerization method described in earlier studies [54,80,81] with variable molar ratios of DPC (crosslinker) and β-CD (selected polymer) as 2:1, 4:1, 6:1, 8:1, and 10:1. The obtained placebo NSs were subjected to drying (at 40 °C) and subsequently kept inside a desiccator.

The drug solubilization capacity of both placebo NSs (across all batches prepared) and β-CD were examined. Briefly, an excess quantity of SLZ (20 mg) and a specific quantity (20 mg) of prepared NSs or β-CD were taken in screw-capped scintillation vials containing water (20 mL). The mixture was shaken for 24 h at 25 °C ± 1 °C using a mechanical stirrer. The resultant mixture was then centrifuged (10 min at 10,000 rpm), and the supernatant was collected. Using DMSO as a solvent, the drug was extracted from this supernatant [72]. SLZ was analyzed in supernatant solutions at λ_max_ = 360 nm by UV.

SLZ was encased in placebo NSs by lyophilization, as described earlier [54,81]. The prepared SLZ-encased NS batches were named SLZ-NS2, SLZ-NS4, SLZ-NS6, SLZ-NS8, and SLZ-NS10 on the basis of molar ratios of polymer and crosslinker utilized for preparing the NSs.

To assess the SLZ quantity in prepared batches, a weighed amount of SLZ-loaded NSs were dispersed in DMSO, sonicated for 10 min, and diluted accurately. The presence of the polymer and its NSs were not found to affect the SLZ absorbance in DMSO and phosphate buffer (pH 5.4). The SLZ amount in the batches was analyzed using a UV spectrophotometer (at 360 nm). Loading capacity, along with the encapsulation efficiency of the drug, was computed by the previously used equations [72]. Briefly, the accurately weighed quantities of all batches of SLZ-loaded CDNS were dispersed in DMSO, followed by sonication (10 min) to break the nanocomplexes, and diluted appropriately. Further, the fabricated samples were assessed by UV spectrophotometry (at 360 nm) to determine the concentration of SLZ. The SLZ loading capacity and encapsulation efficiency were computed as follows:

$$Drug\;loading\left(\%\right)=\frac{weight\;of\;SLZ\;in\;nanosponges}{weight\;of\;nanosponges}\times 100$$

$$Entrapment\;efficiency\left(\%\right)=\frac{weight\;of\;SLZ\;in\;nanosponges}{weight\;of\;SLZ\;feed\;initially}\times 100$$

Particle size and PDI (polydispersity index), along with zeta potential, were recorded using a Malvern Zetasizer Nano (Malvern Instruments Ltd., Malvern, Worcestershire, UK) [72].

Based on solubilization, loading, and encapsulation results, SLZ-NS4 was selected for characterization studies such as FTIR, PXRD, TGA, DSC, FE-SEM, and TEM by following standard protocols [72,82]. The surface morphology was investigated using FE-SEM and TEM. FTIR spectra and PXRD of SLZ and SLZ-NS4 were obtained using a Perkin–Elmer system (Spectrum BX spectrophotometer, Waltham, MA, USA) and X-ray diffractometer (Miniflex-II), Rigaku, The Woodlands, TX, USA, respectively. DSC of SLZ and SLZ-NS4 was performed using a DSC device (Discovery DSC25 series) with a refrigerated cooling system 90 (RCS 90), TA Instruments, New Castle, DL, USA, and TRIOS software, version 5.1, following standard procedures according to the literature [72]. SLZ and SLZ-NS4 were assessed for thermal analysis by an EXSTAR thermo gravimetric/differential thermal analyzer (SII 6300 EXSTAR, Tokyo, Japan).

### 3.5. In Vitro SLZ Release

For release studies, SLZ (5 mg) or SLZ-NS4 having a similar amount of drug was dispersed in release media (phosphate buffer saline, PBS; 2 mL) and packed properly in an activated dialysis membrane (molecular weight cut off: 12,000 to 14,000 Da. Pore size: 2.4 nanometers). This membrane was then suspended using USP type II dissolution equipment (TDT-08L Electrolab, Mumbai, India) in 200 milliliters of release fluid (PBS; pH 5.5) at 37 °C and 50 rpm. To maintain the sink condition, the same volume (5 mL) of fresh-release medium was added after sampling at predetermined intervals for 24 h. Samples taken (5 mL) out were analyzed using a UV-visible spectrophotometer. To determine the release behavior of SLZ from its dispersion and selected SLZ NSs, release kinetics were assessed. The outcomes of in vitro SLZ release were calculated following the formulae described elsewhere [41,72,83].

### 3.6. Cytocompatibility of SLZ-NS Employing THP1 Cell Lines

The viability of human leukemia monocyte cells (THP1 cells) was assessed using MTT assays. For this, cells (5 × 10^3^cells/well) were seeded into 96-well plates, followed by incubation at 37 °C and 5% CO_2_ for one day. Subsequently, SLZ and SLZ-NS4 (62.5–1000 µg/mL) were added to cells and washed twice using PBS. Each well was properly filled with 20 µL of MTT solution and allowed to incubate at 37 °C. After 4 h, 100 µL DMSO was mixed into each well, and its absorbance was recorded (λ_max_ = 570 nm) employing a Bio Tek Epoch 2 micro plate reader (Agilent, Palo Alto, CA, USA). The percentage of viable cells was calculated in accordance with protocol. Untreated THPI cells returned to 100% normal [76].

### 3.7. SLZ-NS-Embedded Hydrogel Fabrication and Characterization

For the preparation of drug-loaded Carbopol gel (1% *w*/*v*), SLZ-NS4 (having an equivalent amount of SLZ 1% *w*/*v* and SLZ 2% *w*/*v*) and SLZ (1% *w*/*v* and 2% *w*/*v*) were embedded in the hydrogel with continuous stirring to obtain homogeneous SLZ-NS-HG and SLZ-HG, respectively. The viscosity, spreadability, and texture profile of plain Carbopol hydrogel (*p*-HG), SLZ-NS-HG, and SLZ-HG were assessed. The viscosity of all hydrogel samples was assessed using a Brookfield viscometer, Middleborough, MA, USA (model DV-E) at 25 °C [71]. The spreadability of prepared samples was assessed by the glass plate method, as reported earlier [71]. The spreadability of prepared plain hydrogel, SLZ hydrogel, and SLZ-CDNS14 hydrogel was assessed by placing a sample (0.5 g) in a 1 cm circle over glass plates. Different weights, i.e., 15, 20, 30, 50, 70, 100, 150, and 200 (g) were allowed to rest on the formulations, respectively, for 1 min, resulting in the spreading of the gel. The extension was determined with a linear scale. All hydrogel formulations were subjected to texture analysis using a TA-XT2i Texture analyzer, Stable Micro Systems Ltd., Godalming, UK.

### 3.8. Safety Assessment and Mechanistic Investigation of SLZ-NS4 Hydrogel

To evaluate the safety behavior of SLZ-HG and SLZ-NS-HG, the HET-CAM technique was employed. For this assay, Humburg chicken eggs that were fertilized by incubating for 10 days (65% RH and 37.3 °C) were used. The irritation behavior of SLZ-HG and SLZ-NS-HG was assessed by employing a previously reported procedure [84]. NaOH solution (0.1 N) was used as positive control, while SLS (1% *w*/*v*) and normal saline were used as negative control [84]. The irritation score was computed by using the equation:

(1) $$Irritation\;score\;(IRSc)=\frac{5\times \left(301-h\right)}{300}+\frac{7\times \left(301-v\right)}{300}+\frac{9\times \left(301-c\right)}{300}$$

where *c* = coagulation time, *v* = vasoconstriction time, *h* = hemorrhage time. From this equation, IRSc was obtained for every test sample, and the lesions were categorized as severe irritant (9 to 21), moderate (5 to 8.9), slightly irritant (1 to 4.9), and non-irritant (0 to 0.9) [84,85].

The FITC-NS-based hydrogel was fabricated as per the previously reported procedure [86,87]. Using FITC dye, the process and extent of skin penetration of dye-loaded NSs were evaluated using confocal laser scanning microscopy (Olympus FV1200, Olympus, Japan). The Swiss albino mice’s dorsal skin was topically treated with FITC-loaded NS hydrogel for a full day, and then they were sacrificed. After that, the dorsal skin was removed and cleaned with PBS (pH 7.4). Skin samples were then evaluated to observe the distribution of dye-loaded NSs from samples in skin annexes [88].

### 3.9. In Vivo Anti-Psoriatic Studies of SLZ-NS Hydrogel

In vivo anti-psoriatic studies were performed employing the tail model and IMQ-induced psoriasis mouse model.

#### 3.9.1. Mouse Tail Model

The anti-psoriatic activity of developed nanogels was performed following a previously designed protocol [71]. All sample formulations were applied on Swiss mice tails (once daily) for two weeks to various groups (Table 6). Mice were euthanized, and their tails were cut off 24 h after the last hydrogel treatment. The tail samples were longitudinally dissected, and cartilage was extracted for pathological examinations [64]. After being cleaned with saline solution, skin samples were treated with 10% neutral buffered formalin. Skin samples were embedded in paraffin (for sectioning) and treated with xylene and isopropanol before being stained with hematoxylin–eosin dye. The acquired samples were inspected under a microscope to check for granular layer and epidermal changes in the mouse tail. In the end, the earlier reported equations were used to investigate the percentage of orthokeratosis and drug action [89].

#### 3.9.2. IMQ-Induced Psoriasis Mouse Model

The anti-psoriatic potential of SLZ-HG and SLZ-NS-HG (SLZ-NS4 hydrogel) was evaluated using this model. For inducing psoriasis, IMQ cream (50 mg) was topically applied daily (once) on the dorsal skin (shaved) of Swiss mice. Skin thickness, erythema, scaling, and PASI were among the symptoms utilized to assess the degree of induced psoriasis over the course of seven days. A measuring scale ranging from 0 to 4 (0—none,1—mild, 2—moderate, 3—severe, and 4—very severe) was selected for investigating these symptoms [90]. Using the previously mentioned scoring system, a decrease in the intensity of these symptoms was seen, as well as a PASI score. Following euthanasia, mouse skin samples were gathered and treated with 10% neutral buffered formalin. Skin tissues were treated in accordance with the usual procedure following fixation and embedded in paraffin [91]. Using a microtome (Yorco Semi Automatic Microtome, Ghaziabad, India), tissue sections (5 µm thick) were cut, and for histopathological analysis, they were stained with hematoxylin and eosin.

### 3.10. Oxidative Stress and Antioxidant Enzyme Estimation

Swiss mice’s back and tail skins, which were kept in a freezer (−80 °C), were prepared for biochemical examination. The samples were accurately weighed, mixed with PBS, and homogenized for one minute. The resulting homogenate was further centrifuged (15 min at 7000 rpm). A biochemical examination was performed on the supernatant [89,90].

MDA, a byproduct of lipid peroxidation, and antioxidant enzymes (SOD, NO, and GSH levels) were measured using homogenates in order to detect any changes in the antioxidant balance in the tail of Swiss mice. The Biuret method was used to assess the soluble proteins in the homogenate, using bovine serum albumin as a reference [92]. An assay was used to measure the amount of MDA in homogenate samples where the MDA and thiobarbituric acid interacted to create an adduct (1:2). This adduct formation was evaluated by spectrophotometer (λ_max_ = 532 nm) [93].

Nitrite accumulation confirms NO production, and accumulation can be determined with Greiss reagent [94]. To quantify the NO levels, equal volumes of supernatant (0.5 mL) and Greiss reagent (0.5 mL) were well-mixed at room temperature and then incubated for 10 min (in the dark). Samples were then examined using a UV-visible spectrophotometer (λ_max_ = 540 nm). The sodium nitrite standard plot was used to estimate the amount of nitrite in the sample.

SOD was estimated using a previously described approach [95], which established the decrease in nitro blue tetrazolium by SOD and was quantified by a spectrophotometer (λ_max_ = 560 nm). Hydroxylamine hydrochloride was added to initiate the reaction.

GSH levels were measured following the procedure outlined earlier [96]. Initially, 1 mL of homogenate and 1 mL of 4% *w*/*v* sulfosalicylic acid were combined. After 1 h of incubation (at 4 °C), to facilitate precipitation, the mixture thus obtained was centrifuged (1200× *g* for 15 min at 4 °C). The resulting supernatant (0.1 mL) was mixed with 2.7 mL of phosphate buffer (0.1mmol/L; pH 8.0) and 0.2 mL of Ellman’s reagent. The formation of a yellow color was immediately measured for absorbance at 412 nm by a UV-visible spectrophotometer. The absorbance coefficient of the GSH/mg protein-specific chromophore (1.36 × 10^4^ M−1/cm) served as the basis for the quantification.

### 3.11. Data Analysis

The collected data (average ± SD/SEM) were statistically examined by two-way/one-way ANOVA test, followed by Bonferroni post-tests/Tukey’s post-test for multiple comparisons where appropriate. The software GraphPad Prism (version 5.01, GraphPad software Inc. San Diego, CA, USA) was used. *p* < 0.05 was designated as the significance level.

## 4. Conclusions

In the current study, SLZ was incorporated into NS, which resulted in a significant augmentation of SLZ solubility (up to 15 folds). The physicochemical and structural characterization revealed that SLZ was successfully encapsulated into NS architectures. The MTT assay demonstrated an enhanced cytocompatibility of SLZ-NS4 compared to free SLZ against THP1 cells. In vitro release indicated a slow release of SLZ from NSs (78.34 ± 2.88% SLZ release in 24 h). The incorporation of SLZ-NS4 into Carbopol 934 has a prominent effect in the treatment of psoriasis. The prepared hydrogel demonstrated suitable rheology, texture, and spreadability for topical application. The HET-CAM assay advocated SLZ nanogel safety (IRSc: 0.86 ± 0.15, non-irritant) over pure SLZ gel (IRSc: 5.44 ± 0.56, moderate irritant). The dermal targeting capacity of hydrogel was additionally confirmed in vivo by visualizing the localization of the drug in skin annexes and dermal uptake via the confocal laser scanning microscopy technique. In vivo anti-psoriatic activity (mouse tail and IMQ-induced mouse model) indicated the higher therapeutic potential of the SLZ-NS hydrogels. From the mouse tail model, the relative epidermal thickness, % orthokeratosis, and drug activity were found to be 28.73 ± 1.19, 74.77 ± 1.77, and 62.76 ± 2.99, respectively, for SLZ-NS-HG (equivalent to SLZ 2% *w*/*v*) which was significantly higher with respect to SLZ-HG. From the IMQ-induced mouse model, the reduction in PASI score was found to be 84.86 ± 5.74 for SLZ-NS-HG (equivalent to SLZ 2% *w*/*v*), advocating its effectiveness in psoriasis. Phenotype, histopathology, and biochemical results further revealed profound antipsoriatic activity of SLZ-NS-HG. In a nutshell, it could be concluded that fabricated novel hydrogel could play a promising role in controlling hyperproliferation of keratinocytes and manage psoriasis non-invasively. Additionally, SLZ and SLZ-NS-loaded hydrogel can be explored in-depth for its mechanism of action. The efficacy of this formulation could be further substantiated by conducting multi-centered clinical trials. In addition, commercialization and regulatory approval of SLZ-NS-HG may require additional validation via clinical trials. Thus, it can be concluded that NS-based hydrogels offer a localized effect, providing a potential approach in the treatment of psoriasis infection as an alternative to the systemic options.

## Figures and Tables

**Table 1:** Characteristic properties of fabricated sulfasalazine-loaded nanosponges.

Sr. No.	Batches	β-CD: DPC (Molar Ratio)	Mean Particle Size (nm) ± SD	PDI ± SD	Zeta Potential (mV) ± SD	EE (%) ± SD	LC (%) ± SD
1	SLZ-NS2	1:2	306.77 ± 97.57	0.449 ± 0.289	−11.90 ± 1.81	70.02 ± 2.63 ^c,d^	11.67 ± 0.44 ^c,d^
2	SLZ-NS4	1:4	277.90 ± 40.00	0.255 ± 0.017	−13.74 ± 2.15	86.39 ± 1.89 ^a,b,c,d^	14.40 ± 0.31 ^a,b, c,d^
3	SLZ-NS6	1:6	337.97 ± 35.33	0.291 ± 0.118	−16.31 ± 3.87	79.62 ± 1.49 ^a,c,d^	13.27 ± 0.25 ^a,c,d^
4	SLZ-NS8	1:8	397.83 ± 83.73	0.573 ± 0.104	−16.93 ± 2.44	59.58 ± 1.40 ^d^	09.93 ± 0.23 ^d^
5	SLZ-NS10	1:10	276.73 ± 67.30	0.271 ± 0.061	−15.90 ± 3.50	42.11 ± 1.91	07.02 ± 0.32

Data given here are the average of three trials. Statistics by one-way ANOVA and Tukey’s test. Encapsulation efficiency and loading capacity: (^a^) *p* < 0.001 vs. SLZ-NS2, (^b^) *p* < 0.01 vs. SLZ-NS6, (^c^) *p* < 0.001 vs. SLZ-NS8, (^d^) *p* < 0.001 vs. SLZ-NS10. SLZ-NSs: sulfasalazine-loaded nanosponges. PDI: polydispersity index; EE: encapsulation efficiency; LC: loading capacity.

**Table 2:** Release kinetics sulfasalazine and nanosponge dispersion.

Kinetic Models	Sulfasalazine	Sulfasalazine-Loaded Nanosponges (SLZ-NS4)
Zero-order	0.891	0.864
First-order	0.983	0.967
Higuchi	0.966	0.975
Hixson–Crowell	0.980	0.867
Korsmeyer–Peppas	0.557	0.534

**Table 3:** Report of texture analysis.

Characteristics	Plain HG	SLZ-HG	SLZ-NS-HG
Hardness ± SD (N)	0.63 ± 0.04	0.68 ± 0.04	0.46 ± 0.03
Cohesiveness ± SD	0.94 ± 0.02	0.85 ± 0.02	0.74 ± 0.02
Adhesiveness ± SD (N s)	−4.43 ± 0.29	−3.77 ± 0.06	−4.05 ± 0.19
Springiness ± SD	0.85 ± 0.01	0.94 ± 0.01	0.86 ± 0.018

**Table 4:** Anti-psoriatic potential hydrogel samples.

Sr. No.	Formulations	Relative Epidermal Thickness (%) ± SEM	% Orthokeratosis ± SEM	Drug Activity ± SEM
1.	Plain hydrogel	100.00 ± 0.00	29.80 ± 1.49	0.00 ± 0.00
2.	Marketed formulation	43.70 ± 1.74 ^a,d^	67.38 ± 2.09 ^a,b,c,d^	52.51 ± 3.37 ^a,b,c^
3.	SLZ1% hydrogel	70.50 ± 6.39 ^a,^	45.75 ± 1.86 ^a,^	21.17 ± 3.41 ^a,^
4.	SLZ 2% hydrogel	52.92 ± 3.17 ^a,c^	56.34 ± 2.09 ^a,e^	36.31 ± 3.50 ^a,d^
5.	SLZ-NS 1% hydrogel	39.12 ± 1.69 ^a,d,e^	59.63 ± 1.83 ^a,b^	41.37 ± 2.90 ^a,b^
6.	SLZ-NS 2% hydrogel	28.73 ± 1.19 ^a,b,d,f^	74.77 ± 1.77 ^a,b,c,f^	62.76 ± 2.99 ^a,b,e,f^

Data given here are the average of six trials. Statistics by one-way ANOVA and Tukey’s test. % thickness of epidermis: (a) *p* < 0.001 versus Plain hydrogel, (b) *p* < 0.05 versus Marketed ointment, (c) *p* < 0.01 versus SLZ 1% hydrogel, (d) *p* < 0.001 versus SLZ 1% hydrogel, (e) *p* < 0.05 versus SLZ 2% hydrogel, and (f) *p* < 0.001 versus SLZ 2% hydrogel. % Orthokeratosis: (a) *p* < 0.001 versus Plain hydrogel, (b) *p* < 0.001 versus SLZ 1% hydrogel, (c) *p* < 0.001 versus SLZ 2% hydrogel, (d) *p* < 0.05 versus SLZ-NS 1% hydrogel, (e) *p* < 0.01 versus SLZ 1% hydrogel, and (f) *p* < 0.001 versus SLZ-NS 1% hydrogel. Drug activity: (a) *p* < 0.001 versus Plain hydrogel, (b) *p* < 0.001 versus SLZ 1% hydrogel, (c) *p* < 0.01 versus SLZ 2% hydrogel, (d) *p* < 0.01 versus SLZ 1% hydrogel, (e) *p* < 0.001 versus SLZ 2% hydrogel, and (f) *p* < 0.001 versus SLZ-NS 1% hydrogel. Marketed ointment: Compound dithranol ointment; SLZ hydrogel: Sulfasalazine hydrogel; SLZ-NS hydrogel: Sulfasalazine nanosponge-loaded hydrogel.

**Table 5:** Severity index and psoriasis area in different animal groups.

Treatments	PASI (After Therapy) ± SD	% Reduction in PASI ± SD
IMQ only	3.278 ± 0.134	0.00 ± 0.00
Normal control only	0.222 ± 0.070	93.48 ± 2.06 ^a,b,c,d^
IMQ-CDTH Ointment	0.100 ± 0.086	69.05 ± 3.11 ^a^
IMQ-SLZ-HG 1% *w*/*v*	1.111 ± 0.111	66.67 ± 3.33 ^a^
IMQ-SLZ-HG 2% *w*/*v*	0.733 ± 0.163	77.18 ± 5.02 ^a^
IMQ-SLZ-NS-HG 1% *w*/*v*	0.600 ± 0.125	81.68 ± 3.61 ^a^
IMQ-SLZ-NS-HG 2% *w*/*v*	0.467 ± 0.170	84.86 ± 6.29 ^a^

Data given here are the average of six trials. Statistics by one-way ANOVA and Tukey’s test. (a) *p*< 0.001 (in comparison to IMQ alone), (b) *p* < 0.001 (in comparison to IMQ-CDTH ointment), (c) *p* < 0.01 (in comparison to IMQ-SLZ-HG 1%) and (d) *p* < 0.05 (in comparison to IMQ-SLZ-HG 2%). SLZ-NS-HG: Sulfasalazine-loaded nanosponge hydrogel; SLZ-HG: Sulfasalazine hydrogel; IMQ: Imiquimod-treated only; CDTH ointment: Compound dithranol ointment.

**Table 6:** Groups of Swiss mice [5 groups (*n* = 6)] and treatment protocol.

Animal Groups	Daily Treatment Protocol for Swiss Mice (14 Days)
1	With plain Carbopol hydrogel
2	With dithranol marketed formulation
3	With SLZ-HG (SLZ 1% *w*/*v*),
4	With SLZ-HG (SLZ 2% *w*/*v*)
5	With SLZ-NS-HG (equivalent to SLZ 1% *w*/*v*)
6	With SLZ-NS-HG (equivalent to SLZ 2% *w*/*v*)

## Data Availability

The data presented in this study are contained within the article.

## Supplementary Materials

The following supporting information can be downloaded at https://www.mdpi.com/article/10.3390/ph18030391/s1: Figure S1: (a) Solubilization efficiency of sulfasalazine (SLZ) alone and with β-CD, and prepared nanosponges. (b) Loading capacity and (c) encapsulation efficiency of SLZ in prepared nanosponges, respectively; Figure S2: Field emission scanning electron microscopy of (a) sulfasalazine and (b) sulfasalazine-loaded nanosponges; Figure S3: Transmission electron microscopy of sulfasalazine-loaded nanosponges: Figure S4: FTIR spectroscopy of sulfasalazine and sulfasalazine-loaded nanosponges (SLZ-NS4); Figure S5: Powder XRD analysis of sulfasalazine and sulfasalazine-loaded nanosponges (SLZ-NS4); Figure S6: Differential scanning calorimetry thermograms of sulfasalazine and sulfasalazine-loaded nanosponges (SLZ-NS4); Figure S7: Thermogravimetric analysis of sulfasalazine and sulfasalazine-loaded nanosponges (SLZ-NS4). Table S1: Outcomes of validation of sulfasalazine in DMSO and phosphate buffer (pH 5.4).
