# Supplementary material for: Development and Evaluation of Hydrogel-Based Sulfasalazine-Loaded Nanosponges for Enhanced Topical Psoriasis Therapy
Source: Pharmaceuticals (Basel). 2025 Mar 10;18(3):391. doi: 10.3390/ph18030391 (PMC11944453; doi:10.3390/ph18030391)
Supplement: Supplementary file 1 [file pharmaceuticals-18-00391-s001.zip › pharmaceuticals-3491205-supplementary.pdf]

pharmaceuticals

Supplementary Materials

Development and Evaluation of Hydrogel Based Sulfasalazine Loaded Nanosponges for Enhanced Topical Psoriasis Therapy

Sunil Kumar [1,2], Anroop B. Nair [3,*], Varsha Kadian [1,4], Pooja Dalal [1], Babu Lal Jangir [5], Bandar Aldhubiab [3], Rashed M. Almuqbil [3], Ahmed S. Alnaim [3], Nouf Alwadei [3] and Rekha Rao [1,*]

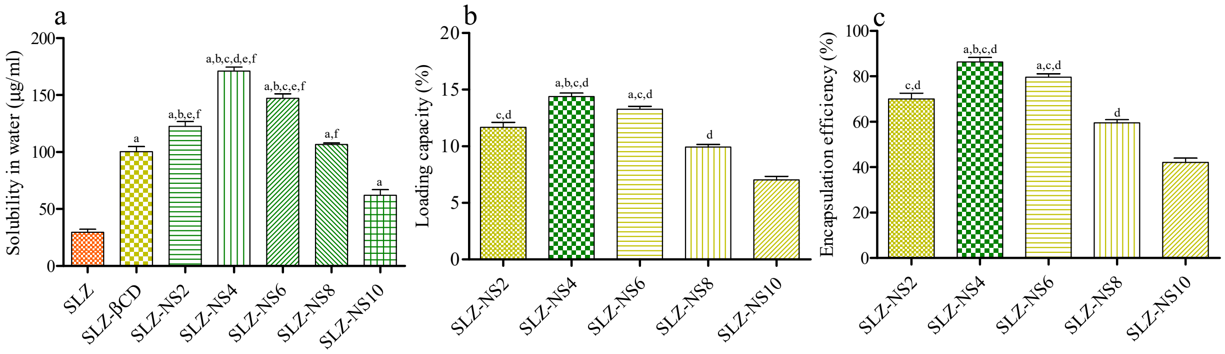

Figure S1. (a) Solubilization efficiency of Sulfasalazine (SLZ) alone and along with β-CD, and prepared nanosponges. **(b)** Loading capacity and **(c)** encapsulation efficiency of SLZ in prepared nanosponges, respectively. Data are in mean ± SD (n=3). Statistical data analysis from one-way ANOVA was followed by Tukey's test for multiple comparisons. For solubilization efficiency: (a) $p<0.001$ vs SLZ, (b) $p<0.001$ vs SLZ-β-CD, (c) $p<0.001$ vs SLZ-NS2, (d) $p<0.001$ vs SLZ-NS6, (e) $p<0.001$ vs SLZ-NS8, (f) $p<0.001$ vs SLZ-NS10. For loading capacity and encapsulation efficiency: (a) $p<0.001$ vs. CSLZ-NS2, (b) $p<0.01$ vs. SLZ-NS6, (c) $p<0.001$ vs. SLZ-NS8, (d) $p<0.001$ vs. SLZ-NS10. SLZ: Sulfasalazine; NS: Cyclodextrin-based nanosponges; β-CD: β-cyclodextrin.

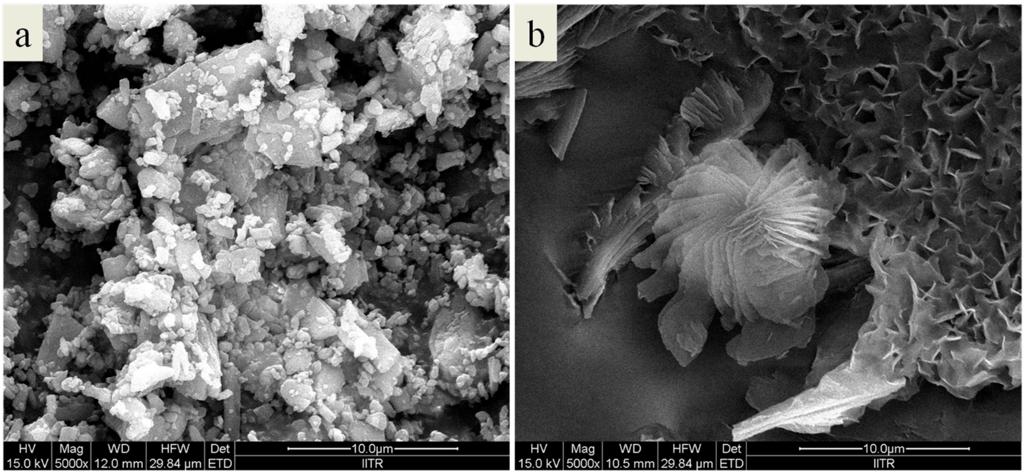

Figure S2. Field emission scanning electron microscopy of **(a)** sulfasalazine and **(b)** sulfasalazine-loaded nanosponges.

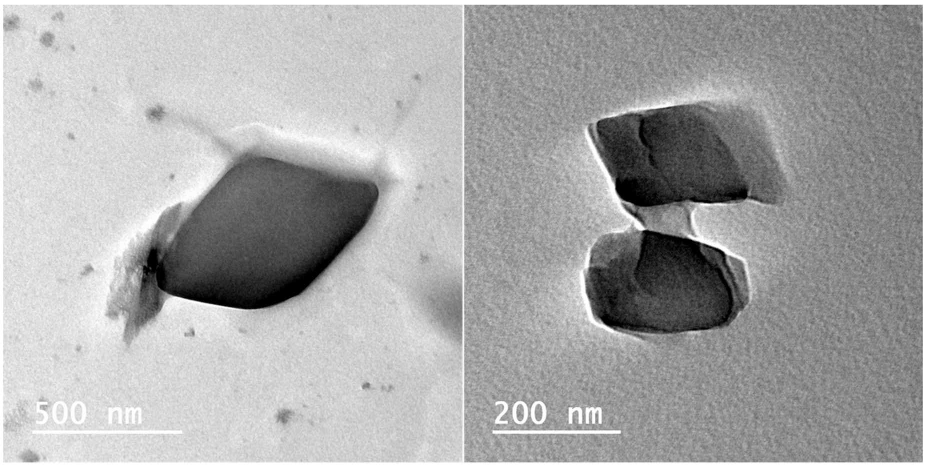

Figure S3. Transmission electron microscopy of sulfasalazine-loaded nanosponges.

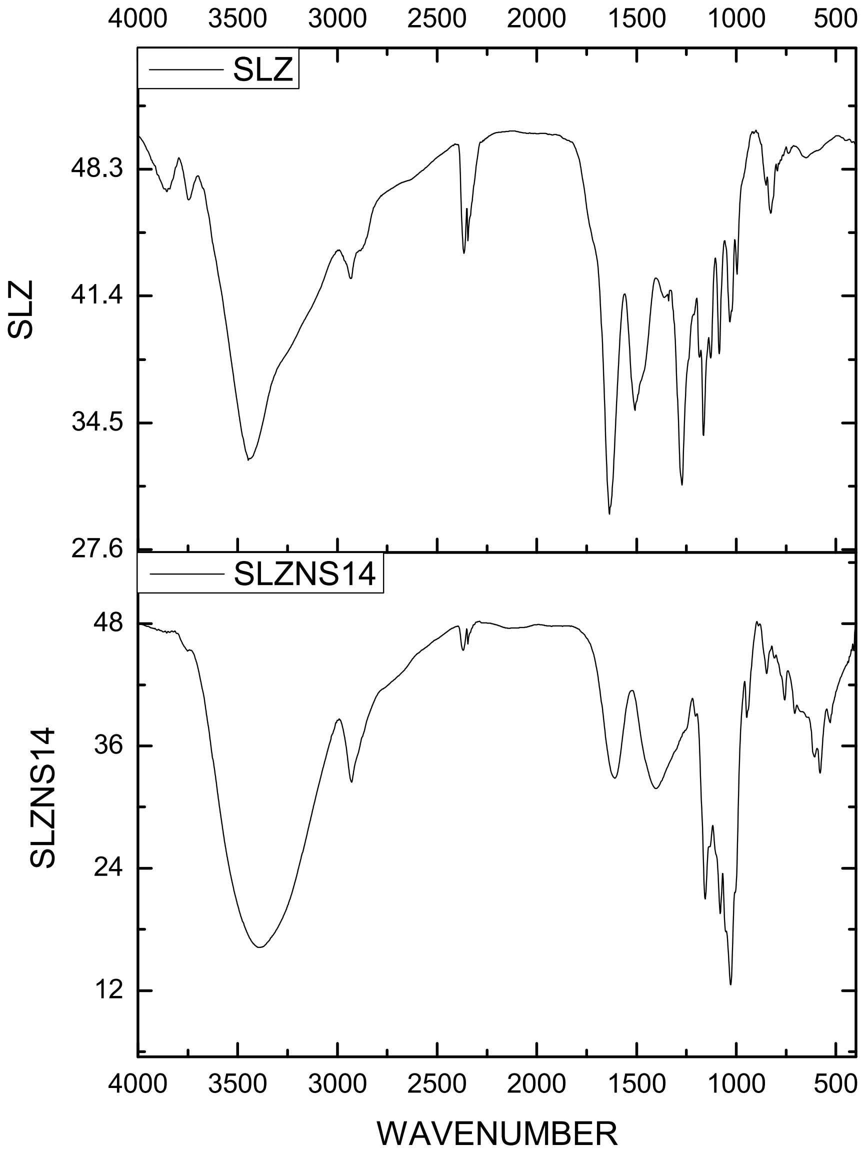

Figure S4. FTIR spectroscopy of sulfasalazine and sulfasalazine-loaded nanosponges (SLZ-NS4).

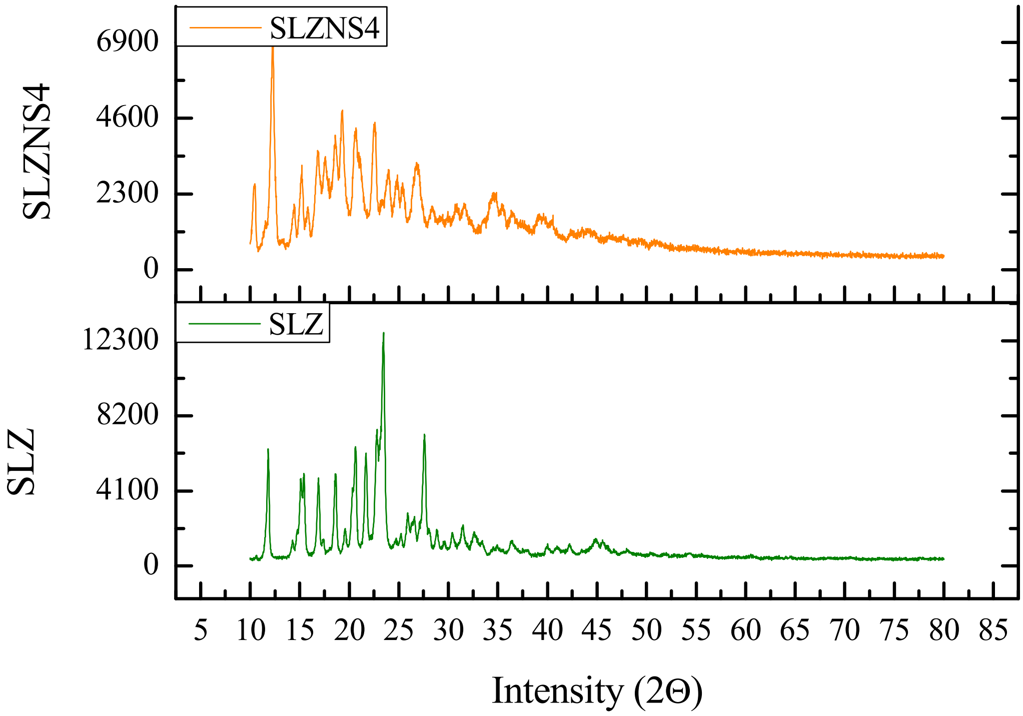

Figure S5. Powder XRD analysis of sulfasalazine and sulfasalazine-loaded nanosponges (SLZ-NS4).

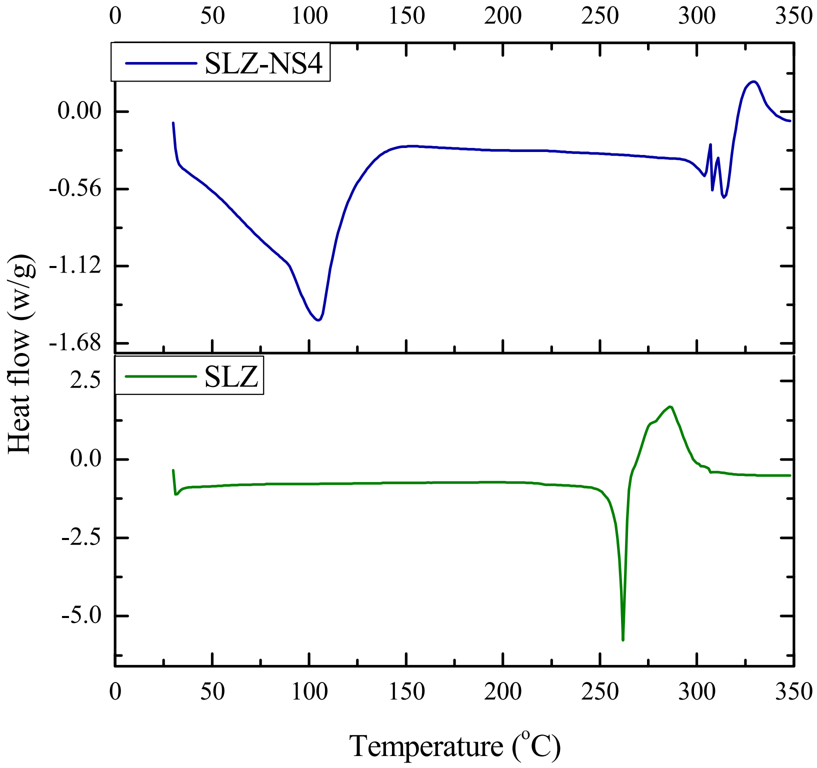

Figure S6. Differential scanning calorimetry thermograms of sulfasalazine and sulfasalazine-loaded nanosponges (SLZ-NS4).

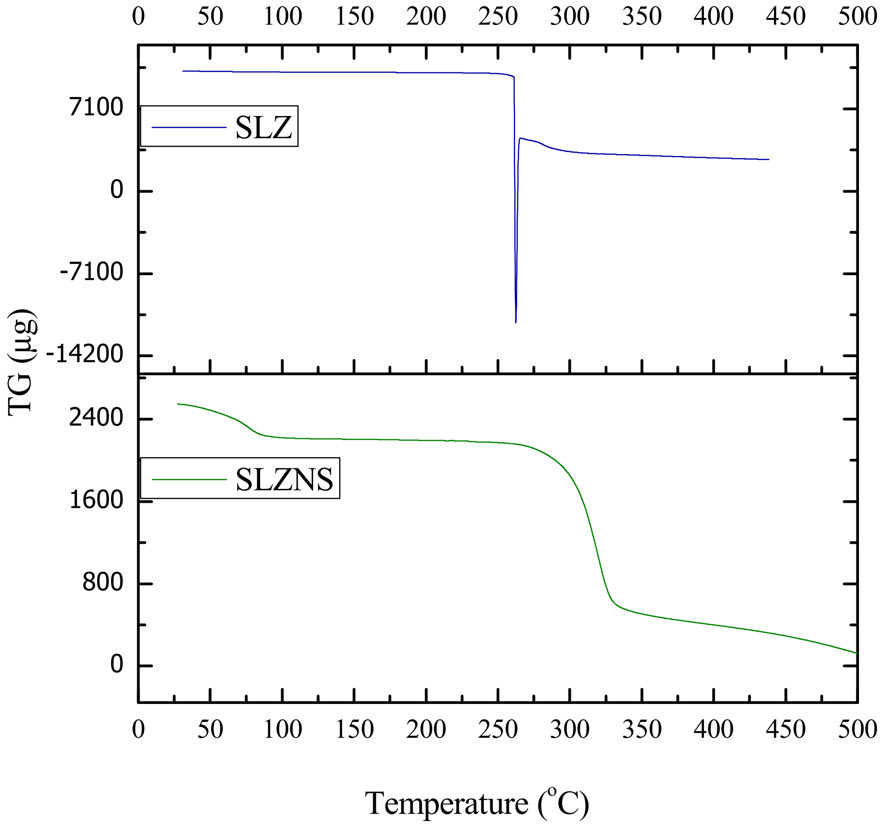

Figure S7. Thermogravimetric analysis of sulfasalazine and sulfasalazine-loaded nanosponges (SLZ-NS4).

Table S1. Outcomes of validation of sulfasalazine in DMSO and phosphate buffer (pH 5.4).

**Factors**	**Solvent**	
	**DMSO**	**Phosphate Buffer**
ʎmax (nm)	360	360
Range (µg/mL)	1–6	1–6
LOD (µg/mL)	0.52	0.012
LOQ (µg/mL)	1.57	0.036
Slope	0.038	0.062
Intercept	-0.006	0.661
Correlation coefficient (r^2)	0.998	0.998
Accuracy (mean % ± RSE)	99.71 ± 0.67	100.66 ± 0.23
Repeatability (%RSD)	0.412	0.168
Intermediate precision (%RSD)	0.641	0.602

RSE: relative standard error; RSD: relative standard deviation.
